# Simulation-based education in urology – an update

**DOI:** 10.1177/17562872231189924

**Published:** 2023-08-10

**Authors:** Angus Ritchie, Maurizio Pacilli, Ramesh M. Nataraja

**Affiliations:** Departments of Paediatrics and Surgery, School of Clinical Sciences, Faculty of Medicine, Nursing and Health Sciences, Monash University, Melbourne, Australia; Departments of Paediatrics and Surgery, School of Clinical Sciences, Faculty of Medicine, Nursing and Health Sciences, Monash University, Melbourne, Australia; Department of Paediatric Surgery and Monash Children’s Simulation, Monash Children’s Hospital, Melbourne, Australia; Department of Paediatric Surgery and Monash Children’s Simulation, Monash Children’s Hospital, 246 Clayton Road, Clayton, Melbourne 3168, Australia; Departments of Paediatrics and Surgery, School of Clinical Sciences, Faculty of Medicine, Nursing and Health Sciences, Monash University, Melbourne 3168, Australia

**Keywords:** paediatric surgery simulation, paediatric urology simulation, simulation-based education, simulation-based medical education, surgical education, surgical simulation, urology education, urology simulation

## Abstract

Over the past 30 years surgical training, including urology training, has changed from the Halstedian apprenticeship-based model to a competency-based one. Simulation-based education (SBE) is an effective, competency-based method for acquiring both technical and non-technical surgical skills and has rapidly become an essential component of urological education. This article introduces the key learning theory underpinning surgical education and SBE, discussing the educational concepts of mastery learning, deliberate practice, feedback, fidelity and assessment. These concepts are fundamental aspects of urological education, thus requiring clinical educators to have a detailed understanding of their impact on learning to assist trainees to acquire surgical skills. The article will then address in detail the current and emerging simulation modalities used in urological education, with specific urological examples provided. These modalities are part-task trainers and 3D-printed models for open surgery, laparoscopic bench and virtual reality trainers, robotic surgery simulation, simulated patients and roleplay, scenario-based simulation, hybrid simulation, distributed simulation and digital simulation. This article will particularly focus on recent advancements in several emerging simulation modalities that are being applied in urology training such as operable 3D-printed models, robotic surgery simulation and online simulation. The implementation of simulation into training programmes and our recommendations for the future direction of urological simulation will also be discussed.

## Introduction

In recent decades, urology training, and surgical education more broadly, has evolved to focus on competency-based education, moving away from the traditional Halstedian apprenticeship-based model of ‘see one, do one, teach one’.^1^ The apprenticeship-based model requires significant time and close supervision to be effective but has been shown to be a valuable method of teaching new skills.^2^ However, recent decreases in working hours for doctors have placed a strain on this method.^3^ In the past 30 years, there has been a global shift towards providing safer working conditions for trainee doctors, led by the reforms introduced by Sir Kenneth Calman in 1993 (the ‘Calmanization’ of surgical training) and the European Working Time Directive in 1998.^4–6^ Working hours for hospital doctors have decreased in a step-wise fashion since this time.^7^ Consequently, there are significantly fewer opportunities for urology trainees to learn technical skills in the operating room.^1^ Concerns regarding inadequate clinical experience have thus led to a growing emphasis for urology trainees to achieve certain competencies in technical and non-technical skills to provide safe and appropriate patient care.^8,9^ A solution that compensates for reduced clinical exposure due to decreased working hours, as well as providing urology trainees the opportunity to achieve competency in clinical skills in a safe environment, is simulation-based education (SBE).

In the aviation industry, simulation has long been used to replicate flight and safety procedures, successfully reducing accident rates to almost negligible levels.^10^ In comparison, in surgery, there is still a very high late readmission rate post-surgery.^11^ While there are likely to be many contributing factors, this may be partly due to inadequate training and the associated learning curve, leading to iatrogenic harm.^12^ Increasingly, incorporating simulation into surgical training may reduce medical errors causing adverse patient outcomes.^10^

In urology training, SBE is a rapidly growing and highly effective form of education.^1^ It allows actual clinical scenarios and skills to be replicated and practised in a safe learning environment. Trainees can therefore learn both the technical and the non-technical skills essential for urological practice in SBE.

The aim of this paper is to describe the key concepts of surgical education and SBE, and to give an overview and update regarding the application of SBE in urology training, focusing on emerging simulation modalities. The fundamental teaching concepts relevant to urology training include mastery learning, deliberate practice, feedback, fidelity and assessment. Clinical educators need to understand and incorporate these concepts into their teaching practice to effectively assist trainees in their learning. This article will therefore address these concepts, in turn, before discussing the simulation modalities available in urology training. These modalities are as follows: part-task trainers (PTTs) and 3D-printed models for open surgery, laparoscopic bench and virtual reality (VR) trainers, robotic surgery simulation, simulated patients (SPs) and roleplay, scenario-based simulation, hybrid simulation, distributed simulation (DS) and digital simulation. Specific examples of simulation relevant to urology will be provided for each modality, with particular focus given to recent advancements in operable 3D-printed models, robotic surgery simulation and online simulation. This article will then address the implementation of simulation-based programmes including the boot camp concept, train-the-trainers (TTT) programmes and structured programme implementation, before addressing our recommendations for the future of SBE in urology.

## Effectiveness and benefits of SBE

In medical education, SBE is an effective method of replicating actual clinical scenarios for educational purposes without involving actual patients.^13^

It is important to note that the vast range of simulation techniques makes it difficult to assess the effectiveness of SBE as a whole, as some simulation modes may be particularly effective, while others do not show particular benefit.^14^ Similarly, SBE is usually incorporated into a larger curriculum that also includes clinical experience, making it difficult to determine the extent to which the SBE component is contributing.^14^ Nevertheless, the evidence evaluating SBE as a teaching method in urology generally shows a strong positive effect on learning outcomes and translation of skills into clinical practice.^1,8,13^

In addition, SBE is an ideal adjunct to compensate for the recent reduction in a trainee’s clinical exposure, allowing efficient learning to take place away from the direct clinical environment.^13^ SBE can be used to replicate rare but serious clinical events, such as emergency urological procedures, to improve a trainee’s preparedness for such emergencies.^15^ These events can be hard to come by in the apprenticeship-based model which relies on the chance of a particular real-life case appearing.^16^ Even when the events do occur in actual clinical practice, they may be considered potentially dangerous for an inexperienced trainee to engage in, further reducing the opportunity for trainees in an apprenticeship-based model to gain experience.^16^

The safe learning environment of SBE also provides trainees with greater confidence and knowledge to apply to actual clinical encounters.^17^ If a trainee is struggling with a particular skill, simulation allows them to practise repeatedly in a safe environment to correct their shortcomings and achieve competency.^16^ This also creates a benefit in terms of patient safety, as inexperienced trainees can learn their skills in a simulation before encountering actual patients.^13^

## Key educational concepts of surgical SBE

### Mastery learning

Mastery learning involves a competency-based approach in which a complex task is broken up into smaller, simpler steps.^18,19^ Trainees must master one step before progressing to the next one. This concept is easily and intuitively applicable to a competency-based model of education such as surgical simulation. For instance, a complex urological operation may be divided into individual components that can be learned progressively until the trainee achieves overall mastery. Mastery learning can also apply to learning non-technical skills including teamwork and collaboration, professionalism and patient interaction.^13^

### Deliberate practice

Deliberate practice refers to the use of a structured and focused approach to achieve a goal.^19^ This requires clear instructions to the trainee regarding the objective of the task, engagement by the trainee, measurable metrics of performance, the opportunity for focused repetition of the task and actionable feedback.^20^ The concept of deliberate practice is closely linked to mastery learning, as the aim is for the trainee to gradually improve through purposeful repetition and then finally achieve mastery in that task. A systematic review and meta-analysis by McGaghie *et al.*^19^ in 2011 found that SBE with deliberate practice was superior to clinical medical education for acquiring clinical skills.

### Feedback

Feedback is ‘any information communicated to the trainee that is intended to modify their thinking or behaviour to improve learning’.^21^ Feedback is an important educational tool, and the safe and structured nature of SBE creates an ideal environment for feedback to be provided. Effective feedback is specific and constructive, evaluating the trainee’s performance up to the current time, how their performance is matching the goals of the simulation and what they can do next to enhance their learning.^22,23^ It can come from different sources such as supervising staff, peers or the simulator, and can be informal and unstructured or structured using a framework. Feedback should be incorporated into the concept of deliberate practice, providing trainees with guidance as they perform focused repetition of a task. Without the provision of feedback at various points in the deliberate practice cycle, competency will not be achieved.

Feedback is generally considered to be one of the most effective components to improve learning performance in SBE.^13,22,24^ Savoldelli *et al.*^25^ performed a randomized controlled trial and observed that a simulation scenario without feedback did not improve the trainees’ skills, but the same scenario with feedback led to a significant improvement in performance. In surgical education, a systematic review by Trehan *et al.*^26^ found that intraoperative feedback enhanced surgical performance and reduced error rates. Feedback is an essential part of not only SBE but also medical education more broadly.

### Simulation fidelity

There is no standard definition for fidelity in SBE, and the word fidelity is often used interchangeably with ‘realism’.^27,28^ Fidelity is often interpreted to mean how closely the simulation matches the appearance of the actual clinical situation that it is replicating.^29^ The term ‘high fidelity’ is therefore commonly applied to simulators that are highly realistic in their physical characteristics.

However, the physical reality of the simulation does not always correlate with learning performance.^28,30,31^ Similarly, a high degree of physical reality often brings more complexity into the scenario.^32^ While this may be appropriate for advanced trainees, for novices and for learning simple individual tasks, this can make the simulation unnecessarily complex.^27^ Consequently, the excessive realism could be distracting and lead to cognitive overload in beginners, potentially hindering their learning.^32^

Dieckmann *et al.*^29^ therefore proposed an alternative framework for simulation fidelity, dividing it into three categories: the physical, conceptual and emotional/experiential attributes of simulation.

The physical aspect refers to the physical equipment and technology available, and how closely it represents reality. In the urological simulation, this may take the form of an anatomically correct 3D model that looks and feels like it is real.

The conceptual model relates to the theories and meaning behind the simulation and how believable these are. Despite the physical differences that may be present between the simulation and reality, the simulation may still be high fidelity if the trainee can make sense of the scenario and understand its value relevant to the clinical situation.^29^

The emotional/experiential aspect focuses on the way the simulation is experienced by the trainee.^33^ This relates to their experience of both the clinical situation represented in the simulation and the simulation as a learning activity. If the trainee’s experience matches the goals of the simulated activity and they are immersed in the scenario, this can also create a high-fidelity simulation.^29^

Most commonly, all three models of fidelity are present in a simulation to varying extents. A simulation that focuses on motor skills may need to rely more on physical fidelity, while a simulation involving diagnostic problems or clinical reasoning may rely more on the conceptual model.^33^

Hamstra *et al.*^28^ further expanded this interpretation of fidelity and suggested that fidelity needs to be redefined, with the aim of the simulation instead being ‘functional task alignment’ (FTA). FTA focuses on what the simulator does (its function), rather than how it appears. The function of the simulator needs to match the task requirements.^28^ For example, an animal tissue sample for learning suturing skills may have low physical fidelity, but for beginners it may provide the best level of tissue responsiveness and confer the skills necessary to achieve the learning objectives of the simulation.

Thus, high fidelity (or the alternative term FTA) more accurately refers to simulated scenarios that are highly effective at achieving their learning objectives. This separates it from realism, which refers to the trainee’s perception of how closely the simulation matches the real clinical environment.^34^ Realism is formed by the combination of all three aspects of fidelity, and acts to promote trainee engagement and learning outcomes.^34^

**Figure 1.**
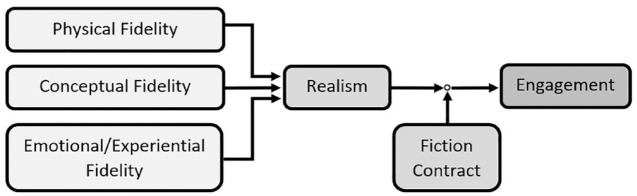
The three modes of fidelity combine to produce the trainee’s perception of the simulation’s realism. The realism of the scenario interacts with the fiction contract to engage the trainee. Source: Based on the work of Rudolph *et al.*^34^

The ‘fiction contract’ also contributes to the trainee’s engagement level.^34^ The fiction contract refers to the agreement between the instructor and the trainee that the simulation has limitations in representing reality (e.g. the mannequin’s skin does not feel real).^33^ The trainee needs to acknowledge this to engage in the simulation. Figure 1 displays the interaction between fidelity, realism and the fiction contract.

### Assessment

Many simulation modes provide reproducible objective assessment measures to evaluate the performance of trainees.^13,16^ These can be formative assessments that can be used to monitor a trainee’s progression and provide ongoing feedback to the trainee, as well as high-stakes tests that are summative assessments of the trainee’s performance. High-stakes tests determine whether the candidate has achieved competence, or has passed the course.^19^ The competency-based model of education can thus be applied successfully in SBE. This allows SBE to be integrated into modern training programmes that require trainees to progressively gain competency in a range of technical and non-technical skills. SBE and SBE-based assessments are integrated into many urology training programmes and courses. For example, the Basic Laparoscopic Urologic Surgery (BLUS) skills programme [adapted from the established Fundamentals of Laparoscopic Surgery (FLS) programme] involves laparoscopic simulation using bench trainers to complete four basic tasks that train essential urologic motor skills.^35,36^ One of these tasks involves applying metal clips onto a 3D-printed renal artery model and dividing the artery with laparoscopic scissors. The task can then be assessed on grading scales using simple, objective measures; time to task completion, complete artery coaptation by clip placement, accurate artery division along the dotted line and the presence of leakage from the two cut ends on completion.^36^

## Limitations of SBE

Despite its significant benefits, there are also several limitations to SBE. Simulation is evident only as a ‘re-creation’ of an actual clinical environment, and there can be significant variability in how realistic and reliable different simulations are.^16^ If certain attributes are missing from the simulation (e.g. the mannequin is not pale or sweating), trainees may view these aspects as unimportant, and may therefore ignore them in the actual clinical environment. This is known as ‘negative learning’, where inadequate technology or simulation design creates a learning experience that could hinder the trainee’s development.^14^ Similarly, trainees in SBE often take shortcuts such as skipping patient consent, or not recording patient data on a blood sample.^14^ When this is transferred to the actual clinical environment, this could lead to these shortcuts being taken with actual patients, setting the standard for unsafe clinical practice.

The artificial nature of simulation can also alter the trainee’s approach to the situation.^37^ The trainee is generally aware that an adverse event is about to occur, or that they are looking for specific concerning features. They may therefore approach the situation with more vigilance than they would in the actual clinical environment.^37,38^ Conversely, there is also the potential for cavalier behaviour by the trainees since they know that the simulation is not real and that no harm can be done to an actual patient.^37^

Another limitation is that simulation equipment can be expensive. This contributes partly to the lower uptake of simulation in low- and middle-income countries compared to high-income countries.^16,39^

Despite these limitations, SBE is generally considered a valuable adjunct to clinical education and the benefits far outweigh the disadvantages.^13,16^

## Simulation methods in urology education

Many forms of simulation are applicable to surgical education including urology. This section will address established methods of SBE for urological training as well as emerging simulation modalities, including recent developments in the areas of 3D modelling and VR training.

### Part-task trainers and 3D-printed models

Learning motor skills is an essential part of urological training and simulation with simple models has long been used as an adjunct to clinical teaching. SBE for open surgical skills in urology incorporates the elements of deliberate practice, mastery learning, and feedback to promote learning.^40^ PTTs are often used to learn these basic technical skills. PTTs are models that are used for repeated practice of a particular skill in isolation. The models can be cadaveric, animal or synthetic based and enable trainees to practise fundamental surgical tasks in a safe environment.^41^ Many basic surgical skills courses are available to teach essential technical skills using simple PTTs and surgical models. An example is practising suturing on porcine tissue or on a synthetic silicone model that mimics human tissue. Specific to urology, PTTs and simple models have been designed and validated for clinical examination and interventions such as male and female pelvic examination, male circumcision and suprapubic catheter insertion.^42–45^ Cadaveric samples have also been used successfully for the simulation of open surgery in emergency urological procedures and renal transplants.^46,47^

In urological training, there are also a growing number of 3D-printed synthetic models available for use as PTTs, as well as for use in laparoscopic and robotic surgical simulation. Improved availability of 3D printers and reduced costs have led to a rising uptake of this simulation modality. Advances in the manufacturing processes of synthetic materials have also enabled the production of highly realistic 3D models, resembling human tissue in appearance and increasingly also in physical properties.^48^ Consequently, 3D prints on which procedures and operations can be performed are now being developed. Examples of 3D-printed models that have been developed for urological training include models for percutaneous nephrolithotomy, partial nephrectomy, laparoscopic pyeloplasty and models for use in robotic surgical simulation.^49–53^ These 3D models are also being used for patient-specific pre-operative simulation, potentially improving intra-operative decision-making and patient outcomes for complex surgeries.^50,54^ In the future, it is likely that high-fidelity synthetic models will largely replace animal and cadaveric models. This is due to the associated logistical, ethical, financial and infection control concerns regarding the use of organic tissue models.^53^

### Laparoscopic bench and virtual reality trainers

The laparoscopic approach is now the favoured approach to many urological surgical interventions such as partial and radical nephrectomy, pyeloplasty and radical prostatectomy (with or without robotic assistance).^55–59^ It provides benefits over traditional open urological surgery while providing equivalent surgical outcomes. The benefits of laparoscopic surgery in urology include reduced postoperative pain, reduced risk of wound infection, shorter hospital stays and earlier return to normal activities for the patient.^55,56^ The skills needed to perform laparoscopic surgery differ from those in typical open surgery in several ways. These are summarized in Table 1.

**Table 1.** Specific skills of laparoscopic surgery that are different from those of open surgery.

Specific laparoscopic surgical skill	Description
The fulcrum effect	This describes the inversion of the surgeon’s movements when using laparoscopic instruments.^60^ If the instrument is moved to the left outside the abdomen, the instrument will move to the right inside the abdomen and *vice versa*. This effect can be confusing to trainee surgeons.
Altered hand–eye coordination and depth perception	Since the surgeon cannot directly visualize the operating field in laparoscopic surgery and instead looks at the monitor, they need to adapt their hand–eye coordination and depth perception.^61^
2D-to-3D realization	In most laparoscopic procedures, the surgeon sees a 2D image on the monitor and needs to be able to convert this to the 3D operating field.^62^
Amplification of movements	The length of the laparoscopic instruments means that movements made by the surgeon’s hands are amplified in the operating field.^60^ Fine motor skills are therefore highly important.
Loss of tactile sensation	In open surgery, the surgeon can directly interact with the operating field and this provides them with more information about the operation.^63^ An example is the surgeon palpating internal organs to locate a tumour. The use of laparoscopic instruments, as well as the distance between the surgeon and the operating field, means that this sensation is greatly reduced in laparoscopy.^63^

Learning these complex motor skills is essential for laparoscopic training. SBE for learning laparoscopic skills has been shown to be highly effective for both two main forms of laparoscopic simulation: bench trainers and VR trainers.^64,65^

Bench trainers are the most common SBE method for learning laparoscopic skills. They consist of a training box with ports through which laparoscopic instruments can be inserted and a screen to see the operating field inside. These features mimic actual laparoscopic surgery and enable urology trainees to practise the techniques that make laparoscopy different from open surgery.^60^ Motion tracking software that monitors instrument movements in the operating field is also commonly incorporated into bench trainers, and provides additional training metrics.^66^ Bench trainers have typically been expensive, but new cheaper models such as the eoSim™ (eoSurgical, Edinburgh, Scotland) are now available, allowing bench trainers to be widely accessible. A study by Hennessey *et al.*^67^ showed that the performance of trainees on this bench trainer correlated with their performance on more expensive bench trainers, validating the eoSim as an effective simulation tool.

VR trainers are another emerging field in laparoscopic simulation. They share similar underlying principles and benefits to bench trainers, but use more sophisticated technology.^68^ The trainee uses laparoscopic instruments which work in a computer-simulated operating field, and this is displayed on a monitor.^69^ This is a virtual environment, as there is no physical operating field. VR trainers, therefore, require sophisticated programming, but can also provide detailed technical performance metrics.^69^

In urology training, various tasks ranging from simple to complex have been proven to be effective for learning simulated laparoscopic skills on bench and VR trainers.^1^ For novice urology trainees, as we have described, the BLUS programme is a validated training model for learning basic skills on bench trainers.^36^ This programme, adapted from the general surgical programme FLS, uses low-cost materials and simple tasks to confer essential technical skills for urological surgery. An example task shared by the FLS and BLUS programme is peg transfer, in which the trainee is required to use the laparoscopic instruments in the bench trainer to place several rings on pegs attached to a board. Despite being a simple model that is far removed from a real operation, this exercise improves the trainee’s abilities of 2D-3D realization, fine motor control and depth perception – essential skills for laparoscopic surgery.^36^ In terms of more complex tasks, as we have described above, many 3D-printed models are becoming available for laparoscopic bench trainers and have been validated for use in urological training.^48^ On VR laparoscopic trainers, simulators and programmes have been validated for procedures such as retroperitoneal radical nephrectomy and robot-assisted radical prostatectomy.^70,71^

### Robotic surgery simulation

Robotic surgery is growing substantially, and consequently, training for robotic surgery is also facing rising demand.^53^ Since the first robot-assisted laparoscopic radical prostatectomy was performed in 2000 using the da Vinci™ (Intuitive Surgical, Sunnyvale, CA, USA), robotic surgery has become an established surgical method in urology.^72,73^ It provides benefits over typical laparoscopic surgery by enhancing dexterity, filtering of physiological tremors and reducing ergonomic fatigue for the surgeon.^73^

The growth in robotic surgery simulation may potentially reduce the emphasis on laparoscopic simulation in the future, particularly in high-income settings, where there is greater access to robotic surgery and robotic simulation. In many low-middle income settings, robotic surgery and robotic surgery simulation are relatively inaccessible due to cost and laparoscopic bench trainers will likely continue to play an important role in training.^74^

Robotic surgery simulation incorporates methods of other simulation modalities into the format of robotic-assisted surgery. This can be either VR simulation or non-VR simulation with physical models.

VR trainers for robotic surgery, such as the da Vinci Skills Simulator™ (Intuitive Surgical, Sunnyvale, CA, USA), have been rated well for their realism and content validity.^12,75^ There is also growing evidence supporting the transferability of skills acquired from VR robotic simulation to actual operations.^76^ However, there is a lack of consensus regarding which tasks and performance metrics are most valuable for VR robotic surgical training and further research regarding standardization is required.^75^ In urology, robotic surgery platforms and VR programmes have been validated for learning basic robotic surgery skills, as well as for complex interventions such as robot-assisted radical prostatectomy, partial nephrectomy and urethrovesical anastomosis.^12,71,77,78^

There is also growing evidence for the efficacy of non-VR trainers in improving clinical robotic surgical performance.^1,74,79^ Similar to laparoscopic bench trainers, training tasks used in non-VR robot-assisted simulation range from simple tasks for novices to more complex tasks involving entire urological procedures.^74,80^ Cadaveric, animal and synthetic models have all been employed in this area. Bertolo *et al.*^81^ utilized fresh frozen cadavers for training basic robot-assisted urology skills, finding a significant post-course improvement in skills among urology trainees.^81^ Animal samples, such as porcine tissue for robot-assisted intracorporeal bowel anastomosis and chicken tissue for robot-assisted urethrovesical anastomosis and posterior muscle-fascial reconstruction, have also been investigated and validated.^82,83^ In terms of synthetic models, these can be simple models or more complex 3D-printed models. Several models have been developed for procedures such as robot-assisted partial nephrectomy, kidney transplantation, radical prostatectomy and urethrovesical anastomosis.^50,84,85,86^

### Simulated participants and roleplay

Simulated participants (SPs) and roleplay are important forms of simulation in which patient actors are used to represent a real patient (or a patient’s relative), allowing the trainee to practise non-technical / cognitive skills such as history-taking, physical examination. SPs and roleplay are a form of this skills training, which is an essential aspect of urological education. Non-technical skills are cognitive and social abilities including decision-making, leadership, communication skills and situational awareness.^87^ SPs are mainly used for improving a trainee’s communication, decision-making and patient-contact skills. The SP generally has a standardized role description, which allows this simulation method to be valuable for summative assessments such as objective standardized clinical examinations.^88^ In addition, the SP can also be another source of valuable feedback for the trainee.^88^

Examples of SPs and roleplay simulation that are relevant to urological practice include gaining consent from a parent for their child’s urological intervention or taking a history from a patient with haematuria.

### Scenario-based simulation

Scenario-based simulation is a structured activity with a timeline of events and clear learning goals that aim to replicate an acute clinical scenario. Team-based simulation scenarios allow trainees to interact within a group as would often occur in a real clinical environment. This aims to confer essential non-technical skills, teach trainees communication skills and coordination within a team, improve team efficiency and reduce errors in an actual clinical situation.^87,89^ Indeed, many events, in which patient safety is compromised in surgical practice, involve non-technical errors such as communication breakdown or poor situational awareness.^87^ Non-technical skill training can also include training to recognize fatigue or distractions, which can be significant factors contributing to technical skill errors and adverse outcomes in surgery.^87^ Non-technical skills are relevant to all domains of urology practice; however, there has typically been significantly less focus on non-technical skills in comparison to technical skill acquisition in surgical training.^90^

Scenario-based simulation is often used to simulate emergency and crisis management situations, and for team-based learning. Example courses include Advanced Trauma Life Support and Advanced Paediatric Life Support. The scenarios often involve full-body mannequins which represent the patient. While practical skills are often incorporated into simulated scenarios, non-technical skills such as situational awareness, decision-making skills and communication skills can also be taught through the scenario. Debriefing and feedback after the scenario is also an essential aspect of the learning process that confers additional educational benefits.^87^

Scenarios can be conducted in purpose-built simulation centres or at the location where the actual scenario would take place such as the operating theatre or hospital ward. In urology training, examples of scenario-based simulations that have been successfully employed are urology trainees practising working as a cohesive team with theatre staff and improving their communication skills during a laparoscopic partial nephrectomy, and urology trainees paired with anaesthetic trainees practising a laparoscopic radical nephrectomy with associated simulated critical events of a vasovagal response to pneumoperitoneum and renal vein injury during hilar dissection.^91,92^

There are currently limited standardized curricula and validated assessment methods for non-technical skill training in urology, particularly in emerging fields such as robotic surgery.^93^

However, the Non-Technical Skills for Urological Surgeons (NoTSUS) has been an effort to develop a standardized curriculum and assessment scale in this area.^94^ This programme, adapted from the Non-technical Skills for Surgeons programme, involves using fully immersive simulation scenarios to train the non-technical skills of urology trainees. The NoTSUS scale was validated in this programme as a reliable assessment of non-technical skills, assessing participants across five domains: communication and team skills, management skills, decision-making, situational awareness and resource skills.^94^ However, the authors also noted that this programme requires further development to target it towards a range of different educational levels.^94^

### Hybrid simulation

Hybrid simulation combines two modes of simulation.^95^ Most commonly this involves combining SPs and PTTs. This aims to replicate actual clinical situations in which technical and non-technical skills are often required concurrently. For example, for a urological procedural skill, a trainee may be required to gain consent from a SP and then perform a task such as urethral catheterization on a synthetic model. This allows the trainee to practise their patient interaction and procedural skills at the same time.

### Distributed simulation

Distributed simulation (DS) refers to the concept of creating an easily portable high-fidelity immersive simulation that can be made available whenever and wherever it is required.^96,97^ Previously, high-fidelity equipment has been expensive and limited to specialized simulation facilities. DS aims to improve accessibility to elements of immersive simulations to a wider clinical community with low-cost and transportable materials.^96^ For example, DS can be used to simulate an operating theatre with an inflatable structure containing an operating table, drapes, a portable light and a photograph of an anaesthetic machine to create an immersive experience. This environment can then be combined with other forms of simulation, such as scenario-based simulations and hybrid simulations allowing trainees to practise team-working skills.

Brunckhorst *et al.*^98^ investigated the use of DS for ureteroscopy skill training, assessing both technical and non-technical skills. They utilized a physical bench trainer model – covered with appropriate drapes – within a fully immersive, portable and inflatable ‘Igloo’ that simulated an operating room environment.^98^ Brunckhorst *et al.*^98^ found that the programme was both feasible and educationally valuable for training technical and non-technical skills. Brewin *et al.*^99^ also validated a DS environment for practising transurethral resection of the prostate.

### Digital simulation: Online, virtual and serious games

Online simulation is an emerging field that involves interactive digital learning. Online learning resources such as interactive educational modules are becoming more available in urological and paediatric surgical training. For instance, Li *et al.*^100^ created a shared online interactive module for all theatre staff prior to a paediatric robot-assisted laparoscopic pyeloplasty. This aims to improve teamwork skills by identifying and providing educational material on key points in the procedure where communication is required.^100^

VR simulation is also becoming more widespread and allows the trainee to be immersed in a digital world that can simulate an actual clinical environment. In surgical education for team training, an example is a VR operating room simulating a surgical crisis scenario.^101^ As we have described above, VR simulators are also utilized for technical motor skill training for laparoscopic and robotic urological surgery. These forms of surgery already involve monitors for viewing the operating field, making VR easily applicable to their training.

Another growing form of digital simulation is ‘serious games’.^102^ These games are interactive and can be used to simulate medical problems requiring clinical reasoning or to simulate common medical procedures in a game-like manner.^102^

Video games (particularly games that are first-person and display a three-dimensional space) that are not specifically designed for laparoscopic training have been shown to be beneficial for technical laparoscopic skills such as improving instrument handling speed and reducing surgical errors.^103–105^ This is likely due to the similarities between video games and laparoscopy such as the need for accurate visuospatial skills and the ability of 2D-3D realization.^106^

Serious games have also been developed that are designed specifically for improving skills such as laparoscopic technical skills and basic life support training.^105^ One such game for laparoscopic training is *Underground*, which was developed by ten Cate Hoedemaker and Grendel Games for the Nintendo Wii U platform (Nintendo Co., Ltd, Kyoto, Japan). This game does not involve medical content, but rather involves a story-based fictional world in which the trainee helps robots escape from an underground mine.^107^ Nevertheless, the game was designed to model the technical skills required for laparoscopic surgery.^108^ The trainee also uses two controllers that are designed and function in a similar way to laparoscopic instruments. Concurrent and construct validity has been proven for *Underground.*^108–110^ However, IJgosse *et al.*^108^ also noted that the performance metrics in this serious game were difficult to evaluate and improvements are still needed in developing a more formal approach to assessing its performance metrics.^108^ Specific serious games for urology education have also been developed for learning urinalysis and urology clinical guidelines.^111,112^

Serious gaming is still an emerging field but is showing promise as an innovative simulation method. It may also increase the trainee’s motivation to practise skills by ‘stealth learning’, which describes when the trainee finds the training so enjoyable that they are unaware of the improvements in their educational outcomes.^113^

## Programme implementation concepts

### The boot camp concept

The boot camp concept has been introduced at various levels from medical students to advanced urology trainees. This concept refers to short, focused and intensive programmes that are often delivered around important transition points in training to enhance learning for trainees entering new clinical roles.^114^ This usually involves simulation training, deliberate practice with formative feedback and a high number of supervising trainers (ideally a ratio of 1 trainer: 1 trainee) to simulate a realistic clinical environment.^114^ Despite being resource intensive, these programmes are becoming increasingly popular and various boot camp programmes have been shown to have a high educational value.^114^ In urology training, Young *et al.*^115^ employed the boot camp concept to deliver a 4-day programme to trainees beginning their urology registrar training.^115^ This programme involved simulation training for a wide range of both technical and non-technical skills relevant to urology practice, successfully improving the knowledge and skills of trainees.^115^ In this way, boot camps have the potential to contribute to patient safety by allowing trainees transitioning into new roles to begin working with increased knowledge and skills.

### Train-the-trainers

TTT programmes involve educating trainers in a particular educational intervention to allow them to then deliver this training to their own trainees. TTT programmes can also include training on the educational methods used to deliver this intervention. This model has been effectively employed in many low-income countries to deliver simulation-based training.^116,117^ Typically, a group from a high-income setting delivers an initial training course to select participants from the low-resource setting, who are then able to become the instructors to train others.^116^

### Structured programme implementation

To implement SBE programmes effectively requires a structured, sustainable and reproducible model.^1^ These programmes should also be embedded within broader curricula, incorporating other educational methods (e.g. direct clinical experience evidently remains an essential part of training).^14^

While some structured programmes are being adapted specifically to urology, such as the BLUS programme for laparoscopic training and the NoTSUS programme for non-technical skills, there is still a need for more urology-specific programmes. Likewise, there is a need for greater standardization of simulation-based curricula and assessments to promote structure within urology training.

## Recommendations

There is a wide range of available simulation modalities within urology. As urological practice advances to focussing more on minimally invasive and robotic surgical methods, simulation is also developing quickly in these areas. Further research should particularly focus on the development of realistic synthetic models, robotic simulation, virtual training and creation of simulators that are accesible for all resource settings.

In addition, more focus needs to be provided to improving and training non-technical / cognitive skills, given that deficiencies in this area account for a large portion of adverse patient outcomes in surgery. There is growing evidence to suggest that scenario and team-based simulations are an effective method for training these skills, so further work needs to be done in this area.

As previously discussed, there also needs to be more research into establishing structured and standardized curricula and assessments in urology training.

## Conclusion

SBE is a highly effective and valuable method of education. Clinical educators must therefore have a solid understanding of the educational concepts described in this article that underpin SBE, as well as the potential applications of SBE for teaching trainees. In urology, simulation is a growing field with many new emerging modalities including 3D-printed operable models, robotic surgery simulation, and online and VR simulation. Simulation in urology is becoming more available, more portable and more advanced. However, ongoing efforts are required to improve SBE in urology, particularly focusing on non-technical skills and standardization of curricula and assessments.

## Declarations

**Ethics approval and consent to participate:** Not applicable.

**Consent for publication:** Not applicable.

**Availability of data and materials:** Not applicable. This is a review article with no original data.
